# Joint exposure to outdoor ambient air pollutants and incident chronic kidney disease: A prospective cohort study with 90,032 older adults

**DOI:** 10.3389/fpubh.2022.992353

**Published:** 2022-09-15

**Authors:** Hongyan Liu, Xian Shao, Xi Jiang, Xiaojie Liu, Pufei Bai, Yao Lin, Jiamian Chen, Fang Hou, Zhuang Cui, Yourui Zhang, Chunlan Lu, Hao Liu, Saijun Zhou, Pei Yu

**Affiliations:** ^1^National Health Commission (NHC) Key Laboratory of Hormones and Development, Chu Hsien-I Memorial Hospital and Tianjin Institute of Endocrinology, Tianjin Medical University, Tianjin, China; ^2^Tianjin Key Laboratory of Metabolic Diseases, Tianjin Medical University, Tianjin, China; ^3^Community Health Service Center, Tianjin, China; ^4^Department of Epidemiology and Health Statistics, Tianjin Medical University, Tianjin, China

**Keywords:** air pollution, chronic kidney disease, cohort study, risk score, PM_2.5_

## Abstract

**Objectives:**

There is paucity of studies to investigate the association between combined and long-term exposure to air pollution and the risk of incident chronic kidney disease (CKD) in older adults.

**Methods:**

A prospective cohort of 90,032 older adults who did not have CKD at baseline were followed up from January 1, 2017, to December 31, 2019. Various pollutant data, including particulate matter with diameters ≤ 2.5 mm (PM_2.5_), ≤ 10 mm (PM_10_), nitrogen dioxide (NO_2_), sulfur dioxide (SO_2_), Ozone (O_3_), and carbon monoxide (CO), from all monitoring stations in Binhai New Area, Tianjin were considered in calculating the mean exposure concentration of each pollutant over 2 years. By summing each pollutant concentration weighted by the regression coefficients, we developed an air pollution score that assesses the combined exposure of these air pollutants. Due to the strong correlation between air pollutants, Principal Component Analysis (PCA) score was also developed. The association between air pollutants and incident CKD in the elderly was analyzed.

**Results:**

A total of 90,032 subjects participated in this study with a median follow-up of 545 days. Among them, 22,336 (24.8%) developed CKD. The HR (95% CI) for air pollution score and incidence of CKD was 1.062 (1.060-1.063) and *p* <0.001 after adjusting for all confounders. The adjusted HRs for the quartile subgroups of combined air pollution score were: Q2: 1.064 (1.013–1.117); Q3: 1.141 (1.088–1.198); and Q4: 3.623 (3.482–3.770), respectively (*p* for trend <0.001). The adjusted HRs for the quartile subgroups of air quality index (AQI) were: Q2: 1.035 (0.985–1.086); Q3: 1.145 (1.091–1.201); and Q4: 3.603 (3.463–3.748), respectively (*p* for trend <0.001). When the risk score was over 86.9, it significantly rose in a steep curve. The subgroup analysis showed that male, younger or exercise were more likely to develop CKD.

**Conclusion:**

Combined air pollution score, AQI, and PCA score were associated with an increased risk of CKD in an exposure-response relationship. Our current results might also provide evidence for developing environmental protection policies.

## Graphical Abstract

The combined air pollutants score for incident chronic kidney disease. 
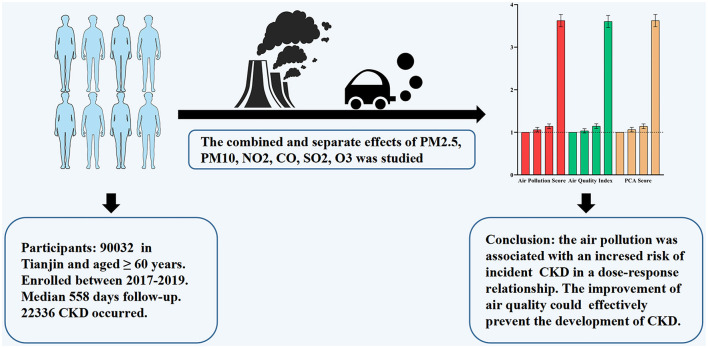

## Introduction

Air pollution is a serious health problem worldwide and has become one of the major environmental problems in China (1). Chronic kidney disease (CKD) is a long-term chronic decline in kidney function and structural kidney damage, negatively impacting the quality of life as it progresses (2, 3). The Global Burden of Disease Study has revealed that CKD was the 16^th^ leading cause of life expectancy loss in 2017, with a 33.7% increase compared to 2007 (4). Furthermore, 17–20% of the worldwide CKD burden could be attributed to PM_2.5_ and was more likely in low- and middle-income countries (5). However, research on the association between long-term exposure to air pollution and the risk of incident CKD in developing countries is still limited. In China, air pollution has become an increasingly prominent problem that cannot be ignored and needs to be addressed as soon as possible.

Several studies have shown that air pollution particles such as particulate matter with diameters ≤ 2.5 mm (PM_2.5_) (6–17), ≤ 2.5 mm (PM_10_) (11, 12), nitrogen dioxide (NO_2_) (10–12, 14, 16), sulfur dioxide (SO_2_) (11, 12, 16), Ozone (O_3_) (12), carbon monoxide (CO) (11, 12), and air quality complex index (AQCI) (18) are associated with an increased risk of kidney outcomes. However, these studies have focused on individual air pollutants. Some studies also lacked variables such as the Air Quality Index (AQI), O_3_, and NO_2_. Therefore, they could not observe combined health effects (7). As individuals are often exposed to a mixture of multiple pollutants, although there is a high correlation between the pollutants, the synergism among pollutants are greater than the sum of the effects of individual pollutants (19). For effective management of multi-pollutant air quality, epidemiological research will be crucial to evaluating pollutant synergism. However, cohort studies focusing on the relationship between combined pollutants and CKD risk are still limited. Moreover, some studies lacked data on dietary, smoking, and drinking habits, which also substantially impact the morbidity of patients (18). Some studies were cross-sectional and, therefore, presented weak causal associations (13). The relationship between variables is more easily observed in large sample cohort studies and less convincing in studies conducted with small samples (12). Additionally, the elderly are more susceptible to decreased kidney function due to air pollutants. However, some studies have not included the elderly (18).

Herein, we used a large cohort population of older people from Tianjin and combined data from air pollutant monitoring sites. Then, we evaluated the association between pollutants and incident CKD, as well as the association between PCA score and air pollution score and CKD. Our current results provided evidence for the environmental risk factors for CKD in older adults based on a large samples and might facilitate the generation of public health policies.

## Methods

### Study design and participants

The Binhai New Area is located in the eastern coastal region of Tianjin. Data for this study were obtained from the Tianjin Chronic Kidney Disease Cohort, including follow-up information from different primary communities in the Binhai New Area, Tianjin, China. About 300,000 people undergo physical examinations each year in this area. Data were recorded in the Tianjin Community Health Service Information System from 2013 to 2019 and were available to downloaded.

**Figure 1:**
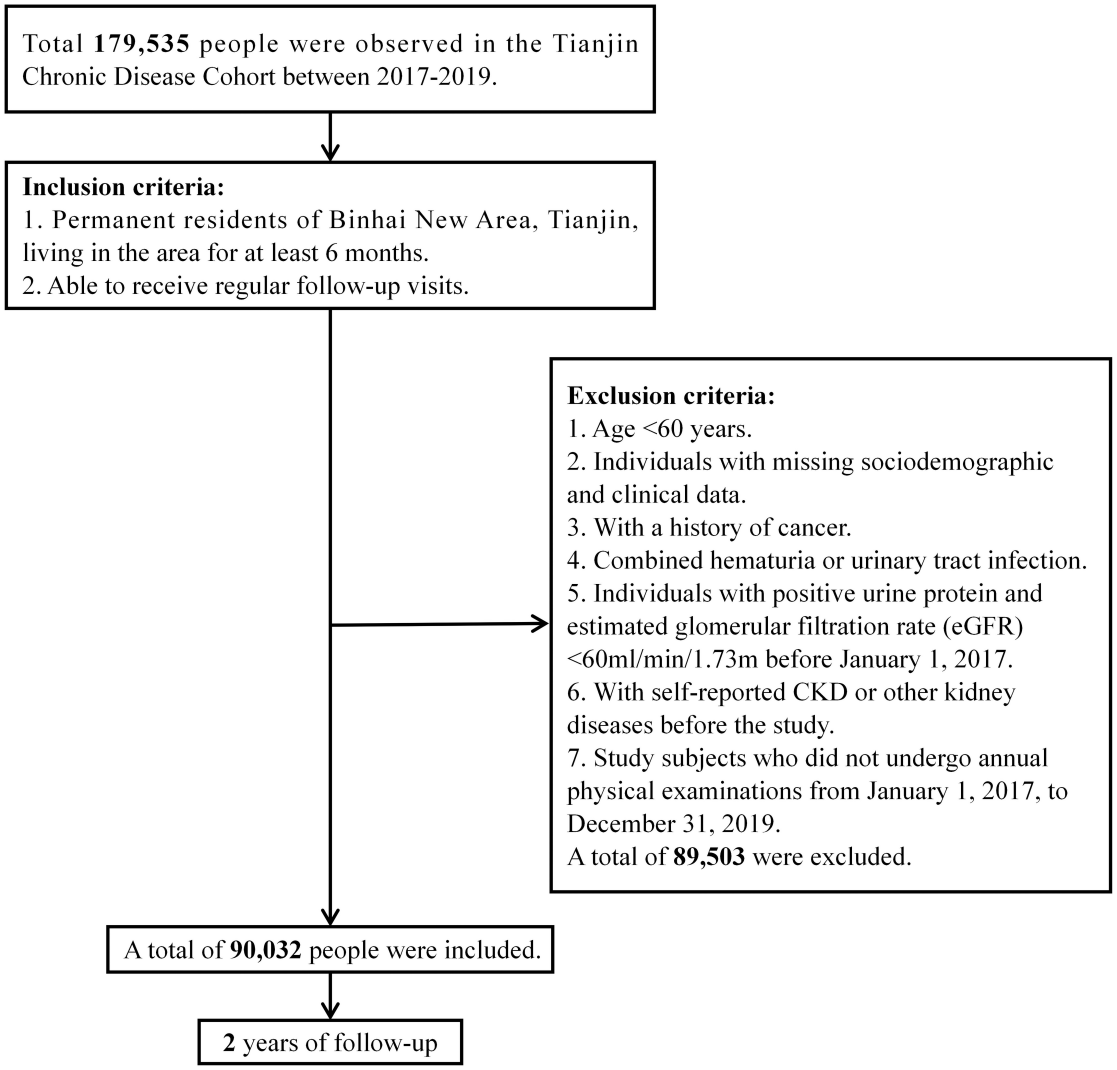
Flow chart of the study.

Inclusion criteria: 1. Permanent residents living in the Binhai New Area, Tianjin, for at least 6 months. 2. Able to received regular follow-up visits. Exclusion criteria: 1. Age < 60 years. 2. Missing sociodemographic and clinical data. 3. History of cancer. 4. Combined hematuria or urinary tract infection. 5. Positive urine protein and estimated glomerular filtration rate (eGFR) < 60 mL/min/1.73 m^2^ before January 1, 2017. 6. Self-reported CKD or other kidney diseases before the study. 7. Did not undergo annual physical examinations from January 1, 2017, to December 31, 2019. A total of 89,503 subjects were excluded. The details of the study process are presented in Figure 1.

A total of 90,032 participants were finally included in the analysis. The study protocol was approved by the Ethics Committee of the Chu Hsien-I Memorial Hospital of Tianjin Medical University and was registered in the Chinese Clinical Trial Registry (ChiCTR1900023701).

### Definition of outcome and stages of chronic kidney disease

Due to the lack of direct diagnostic data for CKD, we evaluated the eGFR using Chronic Kidney Disease Epidemiology Collaboration (CKD-EPI) equation. We used the eGFR as a direct indicator of renal function and proteinuria as a sensitive indicator of renal function, according to the Kidney Disease: Improving Global Outcomes (KDIGO) 2020 Guidelines (20). The outcomes were defined as follows: 1. positive urine protein: daily excretion rate of ≥ 150 mg/24 h, and other confounding factors, such as infection were excluded; 2. eGFR < 60 mL/min/1.73 m^2^. The survival time was defined from baseline to the event occurrence or the end of the investigation. According to international guidelines (20), stage G1: eGFR ≥ 90 mL/min/1.73 m^2^. Stage G2: 60–89 mL/min/1.73 m^2^. Stage G3a: 45–59 mL/min/1.73 m^2^. Stage G3b: 30–44 mL/min/1.73 m^2^. Stage G4: 15–29 mL/min/1.73 m^2^. Stage G5: < 15 mL/min/1.73 m^2^.

### Measurement of air pollution variables

We used data from three ground-based environmental testing sites located in the Binhai New Area, Tianjin between 2014 and 2019, where the surveyed participants had lived for at least 1 year. Seven major air quality indicators, including the AQI, PM_2.5_, PM_10_, NO_2_, SO_2_, O_3_, and CO, were obtained from the national city air quality real-time release platform (http://113.108.142.147:20035/emcpublish/) and the homepage of Wang Xiaolei (https://quotsoft.net/air/). Urban air quality monitoring stations obtain data on the pollutants present in the air through fixed and continuous sampling. Furthermore, the AQI was used to quantitatively describe the air quality condition. The pollutants included in the AQI are SO_2_, NO_2_, PM_10_, PM_2.5_, O_3_, CO. According to the survey design, we calculated the average daily exposure of patient for 2 years before the onset of CKD or cessation of follow-up.

### Definition of joint air pollution score and PCA score

The coefficients for each pollutant were calculated using a multivariable Cox proportional risk regression model. Confounders were adjusted in the composite pollutant model, and joint pollutant scores were calculated to analyze the relationship between joint air pollution exposure and the risk of incident CKD in the elderly. Similar to previous studies (21–24), we used the β coefficients from the final COX model to create the following formula: *Air* pollution score = 0.44 (β1) ^*^ PM_2.5_ + 0.281 (β2) ^*^ PM_10_ + 0.865 (β3) ^*^ NO_2_ + 1.095 (β4) ^*^ SO_2_ + 17.33 (β5) ^*^ CO + (-0.521) (β6) ^*^ O_3._ The air pollution score ranged between 80.85 and 110.77. Then, we distributed the patients into four groups based on the quartiles of the scores.

In order to address the problem of high covariance between air pollutants and their potential interaction, Principal Component Analysis (PCA) was used to analyze these pollutants. The formula of the PCA socre is: PCA score = (0.9603 ^*^ f1 + 0.0227 ^*^ f2 + 0.0099 ^*^ f3)/0.9929. The details are shown in Supplementary Tables 1, 2.

### Measurements of covariates

Baseline data included demographic characteristics (age, gender), behavioral health habits (smoking, alcohol, exercise frequency, diet), history of diseases (diabetes, hypertension), physical examination [body mass index (BMI), waist circumference (WC), systolic and diastolic blood pressure (SBP and DBP)], and laboratory tests [white blood cell (WBC), platelet (PLT), fasting blood glucose (FBG), serum creatinine, eGFR, aspartate transaminase (AST), alanine transaminase (ALT), total cholesterol (TC), and triglyceride (TG)]. The eGFR was calculated using serum creatinine according to the modified MDRD formula. Urine protein was determined using the immunoturbidimetric method. Briefly, urine was collected after cleaning the urethral orifice and vulva before urine retention while avoiding contamination by mixing menstrual blood, leukorrhea, semen, or feces. The first 200 mL of urine was collected early in the morning, and the specimen was delivered within half an hour, with the maximum time not exceeding 2 h. All study personnel were highly trained, and strict quality control procedures were carried out.

### Statistical methods

Indicators with continuous normal distribution are expressed as means (standard deviations - SDs), and a *t*-test was used to compare their differences. Indicators with continuous non-normal distribution are expressed as medians (25th−75th), and the Mann-Whitney *U*-test was used to assess their differences. Categorical indicators were expressed as counts (%), and the χ^2^ test was used to compare them. The HR and 95% confidence intervals (CIs) were estimated using the Cox proportional risk regression model in which several potential confounders were adjusted. Potential confounders were screened by univariate COX analysis. For sensitivity analysis, the HRs (95% CI) was calculated when CKD stages 3–5 were considered as outcome, which indicates a significant decrease in eGFR and < 60 mL/min/1.73 m^2^. Restricted cubic splines provide a powerful way to represent non-linear relationships for continuous independent variables by dividing the observation range of X variables by 3–5 knot points. Thus, we used restricted spline regression to estimate the dose-response relationships between individual pollutants and these scores (AQI, PCA score and the air pollution score) and CKD, which has the flexibility to display non-linear relationships (25). We calculated the inflection point of the cubic splines. Additionally, we conducted a stratified analysis according to gender (male, female), age (60–64, 65–69, 70–74, 75–79, ≥80 years), CKD stages (G1–G5), BMI (<25, ≥25 kg/m^2^), smoking (yes or no), drinking (yes or no), exercise (yes or no), diabetes (yes or no) and hypertension (yes or no) at baseline, and their effect relationships were examined based on interactions effect. The discrimination of these scores was assessed using receiver operating characteristic curve (ROC) and area under the curve (AUC). Calibration was assessed with calibration curves. All analyses were performed using R software (v 4.1.0) and results are expressed by HR and 95% CI. The *p*-values for all tests were two-sided, and a *p* <0.05 was considered statistically significant.

## Results

### Baseline characteristics of the study population

**Figure 2:**
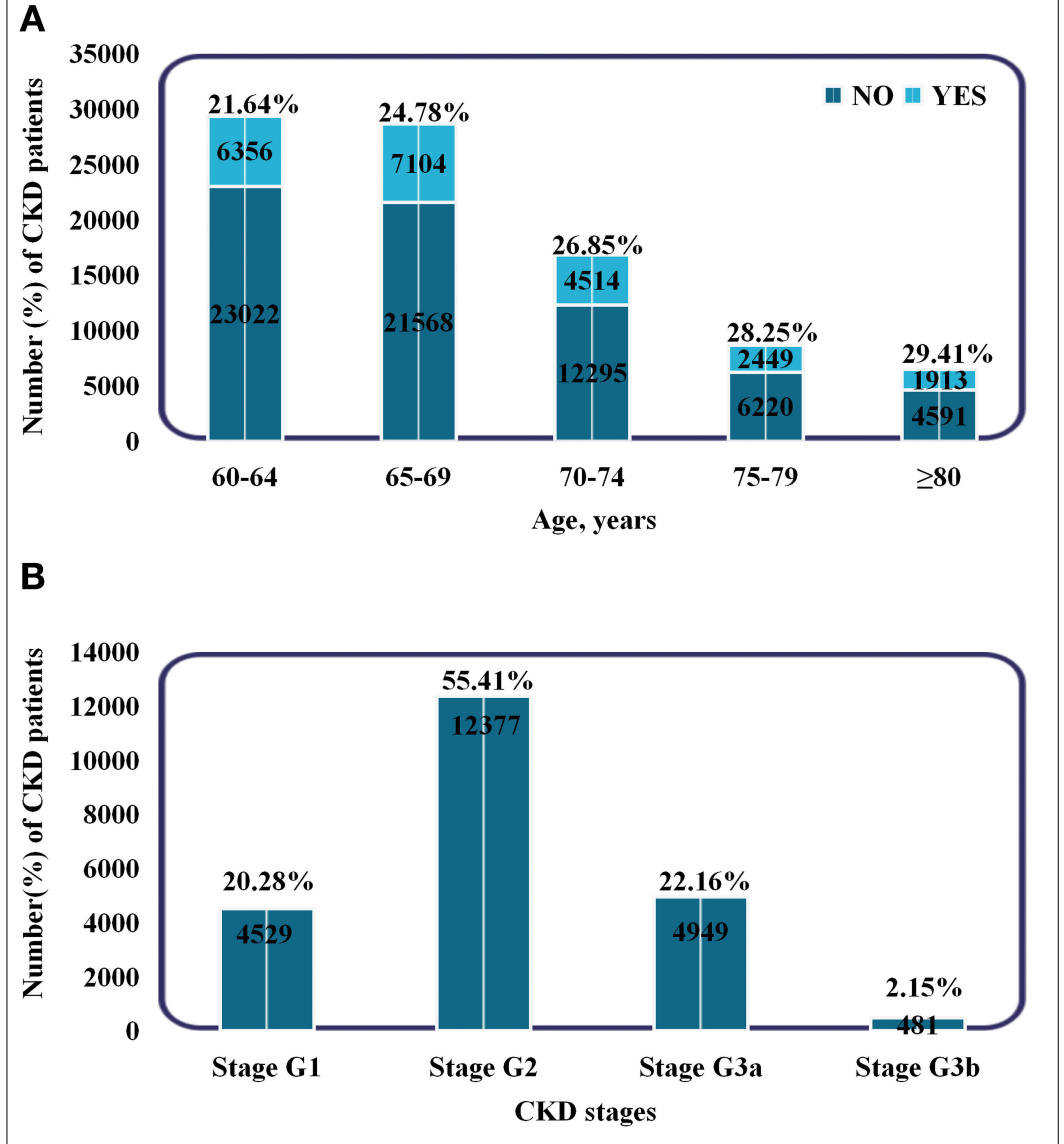
Subgroups of patients based on age and CKD stages. **(A)** Incidence and proportion of CKD patients in different age groups. **(B)** The number and proportion of patients in different CKD stages. Stage G1: eGFR ≥90 ml/min/1.73 m^2^. Stage G2: 60–89 ml/min/1.73 m^2^. Stage G3a: 45–59 ml/min/1.73 m^2^. Stage G3b: 30–44 ml/min/1.73 m^2^.

During a median follow-up of 558 d, a total of 22,336 patients developed CKD among the 90,032 elderly without CKD at baseline. Of these, 4,530 patients had CKD at or above stage 3. The baseline characteristics of the participants according to incident CKD are shown in Table 1. The patients who developed CKD were older (69.07 vs. 68.23 years), females (64.4 vs. 47.3%), and had a higher BMI (25.49 vs. 25.03 kg/m^2^) compared to patients that did not developed CKD. Patients with diabetes and hypertension at baseline were more likely to develop CKD. In the CKD group, the means (SDs) of PM_2.5_, PM_10_, AQI, NO_2_, SO_2_, O_3_, and CO were 58.2 (4.20), 95.0 (6.29), 89.3 (3.95), 47.9 (1.95), 13.8 (1.68), 63.8 (3.25) μg/m^3^, and 1.20 (0.10) mg/m^3^, respectively. In the control group, the means (SDs) were 54.8 (3.12), 90.0 (5.15), 86.1 (3.02), 46.4 (1.50), 12.5 (1.19), 66.1 (2.09) μg/m^3^, and 1.12 (0.07) mg/m^3^, respectively. Moreover, the proportion of CKD increased with aging, and the percentage of people with stage G2 was the highest (48,836, 54.24%) (Figure 2). Additionally, most patients had only a mild decrease in kidney function.

**Table 1:** Baseline characteristics of participants in this study.

**Characteristics**	**Incident chronic kidney disease**		***p* value**
	**No (*N* = 67,696)**	**Yes (*N* = 22,336)**	
Age, years	68.23 (6.22)	69.07 (6.42)	<0.001^a^
60–65	28,151 (77.86%)	8,003 (22.14%)	<0.001^a^
66–70	19,320 (74.92%)	6,468 (25.08%)	
71–75	10,785 (72.89%)	4,011 (27.11%)	
76–80	5,817 (71.35%)	2,336 (28.65%)	
≥81	3,623 (70.47%)	1,518 (29.53%)	
**Gender**
Female	32,042 (47.3%)	14,376 (64.4%)	<0.001^b^
Male	35,654 (52.7%)	7,960 (35.6%)	
BMI, kg/m^2^	25.03 (3.35)	25.49 (4.21)	<0.001^a^
WC, cm	86.00 (8.28)	86.70 (8.77)	<0.001^a^
SBP, mmHg	126.95 (12.3)	128.82 (13.4)	<0.001^a^
DBP, mmHg	77.99 (6.96)	78.12 (7.44)	0.017^a^
Smoking (Yes)	13,773 (20.3%)	3,910 (17.5%)	<0.001^b^
Alcohol (Yes)	12,825 (18.9%)	3,256 (14.6%)	<0.001^b^
Exercise (Yes)	52,209 (77.1%)	17,363 (77.7%)	0.059^b^
**Laboratory tests**
WBC, 10^9^/L	6.03 (1.50)	6.09 (1.57)	<0.001^a^
FBG, mmol/L	5.68 (1.21)	5.94 (1.47)	<0.001^a^
HGB, g/L	140.32 (14.83)	139.06 (14.68)	<0.001^a^
PLT, 10^9^/L	219.53 (54.43)	221.81 (55.45)	<0.001^a^
TC, mmol/L	5.23 (1.20)	5.38 (1.29)	<0.001^a^
TG, mmol/L	1.82 (1.07)	1.91 (1.19)	<0.001^a^
AST, U/L	20.70 (17.00, 25.60)	20.60 (17.00, 25.10)	0.574^c^
ALT, U/L	20.00 (15.00, 26.00)	19.90 (15.00, 26.00)	<0.001^c^
**Comorbidities**
Diabetes (Yes)	9,146 (13.5%)	4,346 (19.5%)	<0.001^b^
Hypertension (Yes)	25,952 (38.3%)	10,462 (46.8%)	<0.001^b^
**Air Pollutants**
PM_2.5_ (μg/m^3^)	54.82 (3.12)	58.20 (4.20)	<0.001^a^
PM_10_ (μg/m^3^)	90.05 (5.15)	95.03 (6.29)	<0.001^a^
NO_2_ (μg/m^3^)	46.38 (1.50)	47.92 (1.95)	<0.001^a^
SO_2_ (μg/m^3^)	12.48 (1.19)	13.81 (1.68)	<0.001^a^
O_3_ (μg/m^3^)	66.10 (2.09)	63.75 (3.25)	<0.001^a^
CO (mg/m^3^)	1.12 (0.07)	1.20 (0.10)	<0.001^a^
AQI	86.12 (3.02)	89.30 (3.95)	<0.001^a^

^a^Independent-samples *T*-test.

^b^Chi-square test.

^c^Mann-Whitney *U*-test.

Normally distributed data are expressed as mean and SD, non-normally distributed data are expressed as median and quartiles, the rest are expressed as counts and percentages. WC, waist circumference; SBP, systolic blood pressure; DBP, diastolic blood pressure; PM_2.5_, particular matter with aerodynamic diameter ≤ 2.5 mm; PM_10_, particular matter with an aerodynamic diameter ≤ 10 mm; NO_2_, nitrogen dioxide; SO_2_, sulfur dioxide; O_3_, Ozone; CO, carbon monoxide; FBG, Fasting blood glucose; HGB, Hemoglobin; PLT, Platelet; WBC, White blood cell; AST, Aspartate transaminase; ALT, Alanine transaminase; TC, Total cholesterol; TG, Triglyceride.

### The relationship between air pollutants and risk of incident CKD

**Figure 3:**
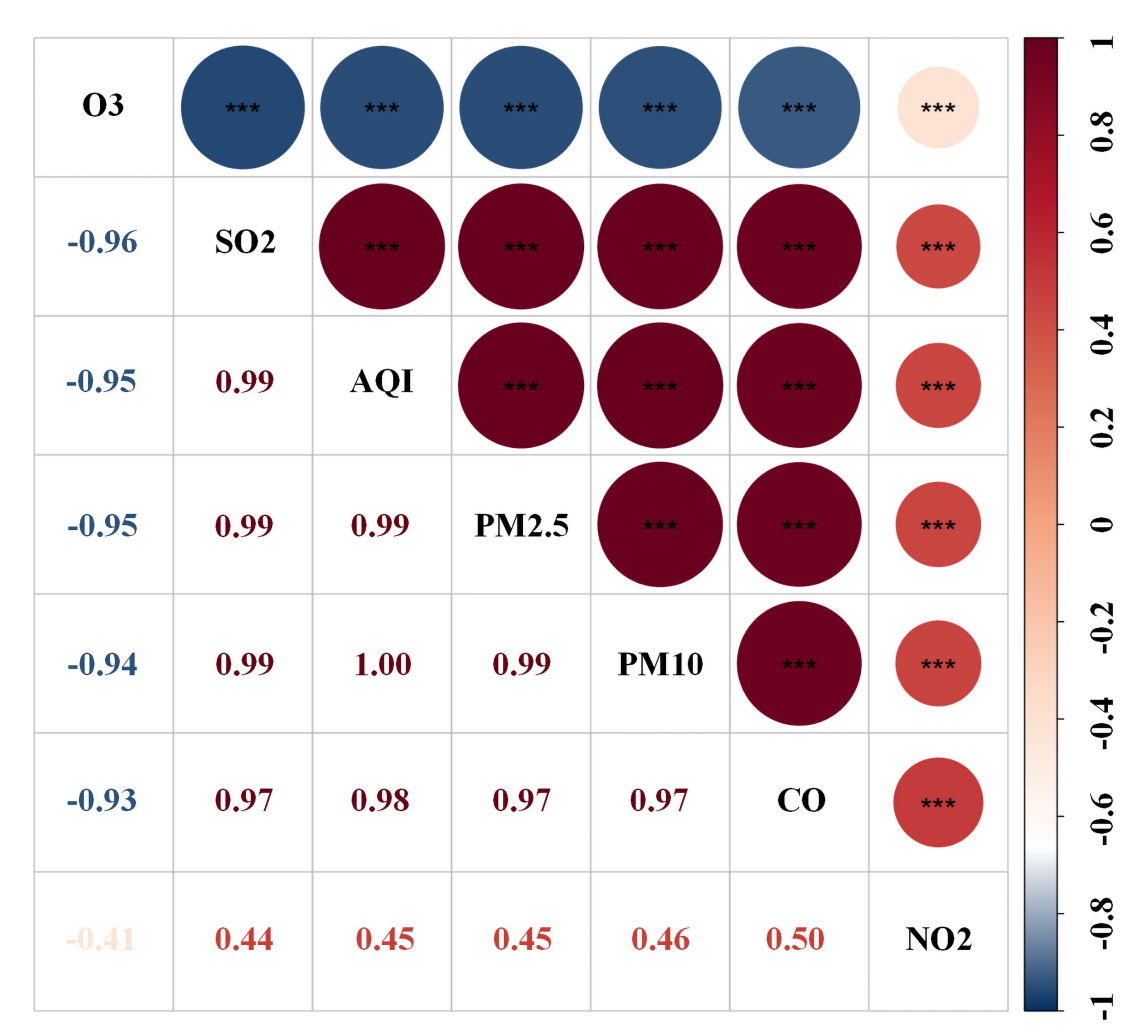
Spearman correlation between air pollutants.

The Spearman correlation analysis suggested a strong relationship between these pollutants (Figure 3). The HR and 95% CI were calculated separately for each pollutant quartile group and are presented in Table 2. As the quartiles increased, the risk of developing CKD significantly increased with the Q1 group as the control. The univariable Cox regression results showed that PM_2.5_, PM_10_, SO_2_, and CO were associated with an increased risk of incident CKD. Additionally, the correlation between each pollutant and the risk of incident CKD remained significant in model 2 and 3 after adjusting for confounding factors (Table 2).

**Table 2:** HR and 95% CI for air pollution concentrations with the risk of incident CKD in the study.

		**Air pollution concentrations (quartiles)**				***p* for trend**
		**Q1**	**Q2**	**Q3**	**Q4**	
PM_2.5_	Model 1	1	1.056 (1.005–1.109)	1.192 (1.137–1.250)	3.870 (3.721–4.026)	<0.001
	Model 2		1.037 (0.987–1.089)	1.176 (1.121–1.233)	3.713 (3.569–3.863)	<0.001
	Model 3		1.027 (0.978–1.079)	1.143 (1.090–1.200)	3.601 (3.461–3.746)	<0.001
PM_10_	Model 1	1	1.089 (1.038–1.144)	1.193 (1.137–1.251)	3.901 (3.750–4.058)	<0.001
	Model 2		1.070 (1.019–1.123)	1.177 (1.121–1.235)	3.744 (3.599–3.895)	<0.001
	Model 3		1.054 (1.004–1.106)	1.140 (3.477–3.764)	3.618 (3.477–3.764)	<0.001
NO_2_	Model 1	1	0.984 (0.938–1.033)	1.125 (1.073–1.179)	3.697 (3.558–3.841)	<0.001
	Model 2		0.979 (0.933–1.028)	1.117 (1.066–1.171)	3.572 (3.438–3.712)	<0.001
	Model 3		0.975 (0.929–1.024)	1.089 (1.039–1.141)	3.472 (3.341–3.608)	<0.001
SO_2_	Model 1	1	1.019 (0.970–1.070)	1.160 (1.107–1.216)	3.772 (3.627–3.922)	<0.001
	Model 2		1.015 (0.966–1.066)	1.152 (1.099–1.208)	3.645 (3.505–3.790)	<0.001
	Model 3		1.009 (0.960–1.059)	1.124 (1.072–1.178)	3.539 (3.403–3.680)	<0.001
O_3_	Model 1	1	0.305 (0.294–0.316)	0.282 (0.271–0.293)	0.255 (0.245–0.265)	<0.001
	Model 2		0.314 (0.302–0.325)	0.288 (0.278–0.300)	0.266 (0.256–0.277)	<0.001
	Model 3		0.315 (0.303–0.326)	0.295 (0.284–0.307)	0.275 (0.264–0.286)	<0.001
CO	Model 1	1	1.089 (1.036–1.143)	1.200 (1.144–1.259)	3.900 (3.748–4.058)	<0.001
	Model 2		1.069 (1.017–1.123)	1.184 (1.128–1.242)	3.742 (3.596–3.894)	<0.001
	Model 3		1.052 (1.002–1.105)	1.148 (1.095–1.205)	3.615 (3.474–3.762)	<0.001

CI, confidence interval; HR, hazard ratio; PM_2.5_, particular matter with aerodynamic diameter ≤ 2.5 mm; PM_10_, particular matter with an aerodynamic diameter ≤ 10 mm; NO_2_, nitrogen dioxide; SO_2_, sulfur dioxide; O_3_, Ozone; CO, carbon monoxide.

Model 1: Univariable Cox regression for air pollutants. Model 2: Adjusted for age and gender. Model 3: Adjusted for age, gender, BMI, smoking, drinking, exercise, history of diabetes, history of hypertension, WC, FBG, SBP, DBP, WBC, HGB, PLT, TC, TG, ALT, eGFR at baseline.

### Association between the AQI, PCA score and combined score and risk of incident CKD

Based on the results of the multivariable analysis and PCA analysis, we developed air pollution score and PCA score. Then, we evaluated the relationship between the scores and CKD risk. For each one-point increase in the air pollution score, the adjusted HR corresponding to CKD risk was 1.062 (1.060–1.063) (Table 3). The quartile grouping of air pollution scores revealed that the HRs (95% CI) for the Q2, Q3, and Q4 groups were 1.064 (1.013–1.117), 1.141 (1.088–1.198), and 3.623 (3.482–3.770), respectively (*p* for trend <0.001). In addition, when the outcome was CKD stage 3–5, the HRs (95% CI) for the Q2, Q3, and Q4 groups of were 1.035 (0.932–1.149), 1.117 (1.010–1.236), 3.464 (3.191–3.760), respectively (*p* for trend <0.001) (Table 3).

**Table 3:** Hazard ratios and 95% confidence interval for the risk of incident CKD in the study.

	**HR (95% CI)**	***p*-value**	**Q1**	**Q2**	**Q3**	**Q4**	***p* for trend**
**HRONIC IDNEY ISEASE**
**Air pollution score**
Model 1	1.065 (1.063–1.065)	<0.001	1	1.099 (1.047–1.154)	1.193 (1.137–1.252)	3.906 (3.755–4.064)	<0.001
Model 2	1.063 (1.061–1.064)	<0.001	1	1.080 (1.028–1.134)	1.178 (1.123–1.235)	3.749 (3.604–3.901)	<0.001
Model 3	1.062 (1.060–1.063)	<0.001	1	1.064 (1.013–1.117)	1.141 (1.088–1.198)	3.623 (3.482–3.770)	<0.001
**Air quality index**
Model 1	1.178 (1.174–1.182)	<0.001	1	1.072 (1.021–1.125)	1.196 (1.140–1.254)	3.885 (3.735–4.041)	<0.001
Model 2	1.173 (1.169–1.177)	<0.001	1	1.049 (0.999–1.101)	1.179 (1.124–1.237)	3.722 (3.578–3.872)	<0.001
Model 3	1.170 (1.166–1.174)	<0.001	1	1.035 (0.985–1.086)	1.145 (1.091–1.201)	3.603 (3.463–3.748)	<0.001
**PCA score**
Model 1	1.277 (1.271–1.284)	<0.001	1	1.099 (1.047–1.154)	1.193 (1.137–1.252)	3.906 (3.755–4.064)	<0.001
Model 2	1.269 (1.263–1.276)	<0.001	1	1.080 (1.028–1.134)	1.178 (1.123–1.236)	3.749 (3.604–3.901)	<0.001
Model 3	1.264 (1.258–1.270)	<0.001	1	1.064 (1.013–1.117)	1.141 (1.088–1.198)	3.623 (3.482–3.770)	<0.001
**CKD TAGE 3–5**
**Air pollution score**
Model 1	1.114 (1.110–1.118)	<0.001	1	0.999 (0.900–1.109)	1.190 (1.176–1.316)	4.502 (4.149–4.885)	<0.001
Model 2	1.095 (1.091–1.098)	<0.001	1	1.085 (0.978–1.205)	1.384 (1.251–1.531)	4.508 (4.154–4.892)	<0.001
Model 3	1.100 (1.096–1.104)	<0.001	1	1.035 (0.932–1.149)	1.117 (1.010–1.236)	3.464 (3.191–3.760)	<0.001
**Air quality index**
Model 1	1.202 (1.194–1.211)	<0.001	1	0.964 (0.869–1.070)	1.185 (1.072–1.310)	4.450 (4.103–4.827)	<0.001
Model 2	1.191 (1.183–1.199)	<0.001	1	1.050 (0.946–1.165)	1.374 (1.243–1.520)	4.452 (4.104–4.829)	<0.001
Model 3	1.165 (1.157–1.174)	<0.001	1	1.019 (0.918–1.132)	1.125 (1.017–1.244)	3.461 (3.190–3.756)	<0.001
**PCA score**
Model 1	1.313 (1.299–1.326)	<0.001	1	0.999 (0.900–1.109)	1.190 (1.076–1.316)	4.502 (4.149–4.885)	<0.001
Model 2	1.297 (1.284–1.311)	<0.001	1	1.085 (0.978–1.205)	1.384 (1.251–1.531)	4.508 (4.154–4.892)	<0.001
Model 3	1.247 (1.234–1.260)	<0.001	1	1.035 (0.932–1.149)	1.117 (1.010–1.236)	3.464 (3.191–3.760)	<0.001

CI, confidence interval; HR, hazard ratio. Model 1: Unadjusted. Model 2: Adjusted for age and gender. Model 3: Adjusted for age, gender, BMI, smoking, drinking, exercise, history of diabetes, history of hypertension, WC, FBG, SBP, DBP, WBC, HGB, PLT, TC, TG, ALT, eGFR at baseline.

Next, we investigated the relationship between the AQI and CKD (Table 3). For every one increase in the AQI, the HR (95% CI) was 1.170 (1.166–1.174) (*p* <0.001). The quartile grouping of AQI revealed that the HRs (95% CI) for the Q2, Q3, and Q4 groups were 1.035 (0.985–1.086), 1.145 (1.091–1.201), and 3.603 (3.463–3.748), respectively (*p* for trend <0.001). Then we investigated the relationship between the PCA score and CKD (Table 3). For every one increase, the HR (95% CI) was 1.264 (1.258–1.270) (*p* <0.001). The quartile grouping of PCA score showed that the adjusted HRs (95% CI) for the Q2, Q3, and Q4 groups were 1.064 (1.013–1.117), 1.141 (1.088–1.198), and 3.623 (3.482–3.770), respectively (*p* for trend <0.001). Furthermore, both the PCA score and AQI showed significant trends of HR when using CKD stage 3–5 as outcomes (Table 3).

**Figure 4:**
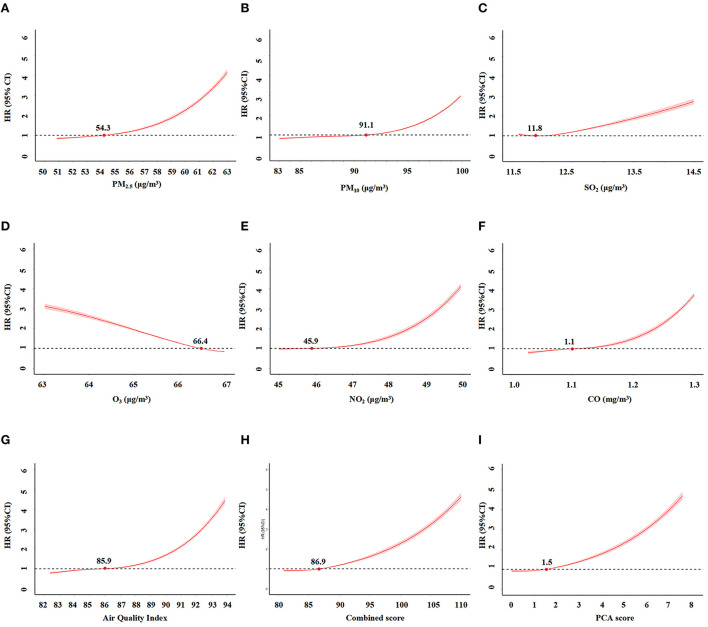
The relationships between air pollution indicators and incident CKD by restriction spline regression. **(A)** PM_2.5_. **(B)** PM_10_. **(C)** SO_2_. **(D)** O_3_. **(E)** NO_2_. **(F)** CO. **(G)** Air Quality Index (AQI). **(H)** combined score of all pollutants. (I). PCA score.

After adjusting for potential confounders, we calculated the non-linear relationship between these scores and CKD risk using restricted spline regression. No significant change in the HR was detected when the score was ≤ 86.9 (Figure 4). Thus, the inflection point for the restricted spline regression was 86.9. The risk of CKD would rapidly increase when the score was over 86.9. Similarly, the inflection point for the PCA score and AQI were 1.5 and 85.9, respectively. Altogether, these results indicated an exposure-response relationship between these scores and the risk of incident CKD. The AUC of AQI, PCA score and combined score were 0.713, 0.715, and 0.715, respectively. Their calibration curves are also similar (Supplementary Figure 1).

### Stratified analyses

**Figure 5:**
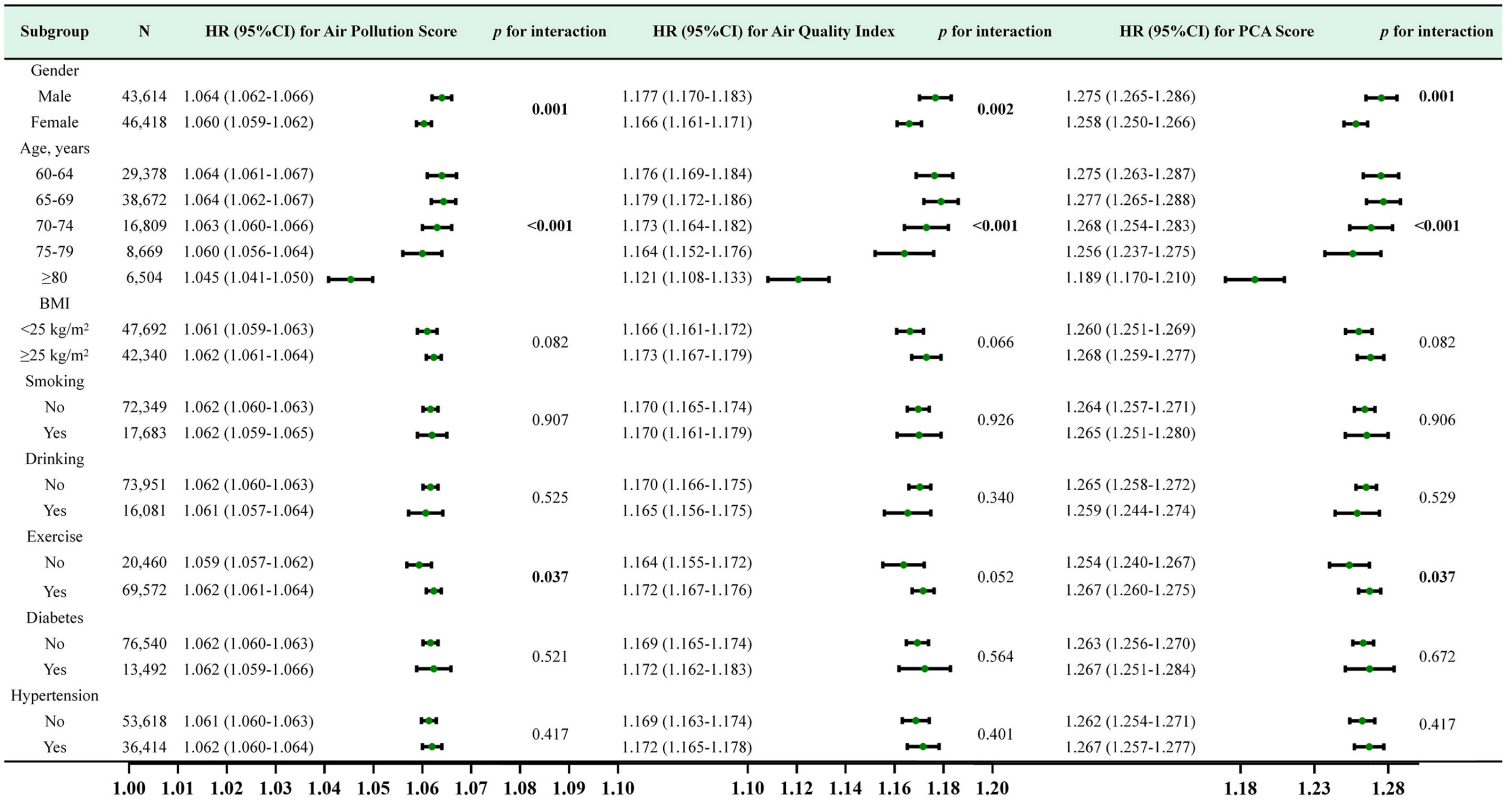
Subgroup analysis and HR (95% CI) for air pollution score, air quality score and PCA score and CKD. All subgroup analyses adjusted for age, gender, BMI, smoking, drinking, exercise, history of diabetes, history of hypertension,WC, FBG, SBP, DBP, WBC, HGB, PLT, TC, TG, ALT, eGFR at baseline.

Furthermore, we conducted stratified analyses for age, gender, BMI, hypertension, diabetes, behavioral habits (smoking, drinking, exercise). For air pollution score and PCA score, The subgroup analysis and interaction effect analyses revealed that male, exercise or younger had a higher risk of incident CKD at the same exposure levels (*p* <0.05). For AQI, The subgroup analysis indicated that male and younger had a higher risk of incident CKD (*p* <0.05) (Figure 5).

## Discussion

Herein, we conducted a large cohort study in northern China and found that long-term exposure to PM_2.5_, PM_10_, SO_2_, and CO were associated with an increased risk of CKD after adjusting for all potential confounding factors. We developed an air pollution score for assessing CKD risk by calculating Cox regression coefficients after adjusting for all potential confounding factors. In addition, due to the strong correlation between air pollutants, PCA score was used to analyze these pollutants. The results revealed no significant change in CKD risk when these scores was below the inflection point. On the other hand, when the risk score was higher, the CKD risk would significantly rise in a steep curve. These results indicated a dose-response relationship between AQI, PCA score and air pollution score and CKD risk, independent of traditional risk factors. Altogether, these results suggested that the prevalence of environmental pollution can significantly effect the kidney health of the elderly population. Our current results provided evidence for further developing public environmental protection policies and encouraging people to make efforts to protect the environment.

Several previous cohort studies have shown that PM_2.5_ (6–10, 13, 15–17, 26), PM_10_ (10, 26–28), SO_2_ (15, 26, 27), NO_2_(14, 26, 27), AQI (18), CO (26, 27) are associated with an increased risk of CKD, consistent with our current findings. Besides, a meta-analysis showed that PM_2.5_, PM_10_, NO_2_, SO_2_ and CO are associated with CKD (28). Some studies have shown that PM_2.5_ and CO are associated with death in CKD patients (11, 17). Li et al. have shown that short- or medium-term NO_2_ exposure are associated with kidney damage (12).

According to the subgroup analyses, we showed that the incidence is higher in women, however, male were more vulnerable to combined air pollution exposure than female. A meta-analysis shows increased risk of progression in men compared with women (29). The EQUAL study also demonstrate faster declines in renal function in men compared with women, even after adjustment for multiple groups of mediators, which may explain why male received more effects (30). Further researches may be needed to explain this pathogenesis. In the current study, patient who are obesity (BMI ≥ 25 kg/m^2^) are more susceptible to the effects of air pollution. The association between increased air pollutant concentrations and CKD prevalence is stronger in the visceral obesity group than normal group (27). According to our findings, people who do not drink alcohol or exercise are more susceptible to the effects of air pollution. As reported by Cui Guo et al., in comparison to no exercise, exposure to equivalent PM2.5 levels is associated with a greater risk of death (31), which means that people who exercise are more susceptible than those who do not exercise. Additionally, younger patients had a higher risk of developing CKD due to air pollution (*p* <0.001), a potential explanation for this is an imbalance in the aging population, with people of higher ages dying from other events. There are no reports on whether alcohol consumption or aging impacts the relationship between air pollution and CKD. It is only reasonable to conclude that non-drinkers and younger are more likely to be affected by air pollution than drinkers.

Although some studies based on satellite spatio-temporal models can accurately predict ground-level PM_2.5_, the measurements of other pollutants, such as SO_2_, NO_2_, PM_10_, AQI, and CO, are missing and cannot be directly determined (8, 9, 14, 27). In this present study, we used measurement data from surface monitoring sites, which comprehends more reliable data than satellite-based assessment. The current literature on SO_2_, NO_2_, PM_10_, AQI, and CO exposure in CKD studies remains limited. Thus, we explored all variables described above. Based on these significant indicators, we established a joint risk score and PCA score, which was more reliable than a single indicator. Besides, this was a longitudinal cohort study, starting with non-CKD patients who were observed for nearly 2 years. In this period, some patients developed CKD, with stronger causal associations compared to those cross-sectional studies (13, 14).

The findings on the relationship between PM_2.5_ exposure and CKD have been inconsistent, with a small number of studies not supporting the conclusion that the exposure increases CKD risk. In a retrospective cohort study (32), the results showed that long-term exposure to air pollution was unlikely to increase CKD risk. Moreover, after unit conversion, the average concentrations of PM10 and CO were 61.7 and 775 μg/m^3^, respectively. However, in our current study, the mean concentrations of PM_10_ and CO was 95.03 and 1,200 μg/m^3^, respectively. Thus, the concentration of PM_10_ was 1.54 times, and CO was 1.55 times higher than in the study of Hwang et al. Additionally, in the study by Hwang et al., 71.9% of the population was under 60 years old, while all patients were over 60 years old in our current research. Hence, we considered that these different results were derived from the different average age of the surveyed population and the concentrations of pollutants. Accordingly, people over 60 years old can have CKD due to greater sensitivity to air pollution. These contradictory results emphasized the necessity to increase the studies in areas with high air pollution.

To the best of our knowledge, this was the first study to assess the risk of CKD in older adults from air pollution exposure using score models based on a large cohort. Additionally, the results were still robust after adjusting for confounders. Moreover, it is possible that certain components play a major role, we are exposed to the air as a whole and cannot be exposed to one specific component alone, while our calculations showed that most component was significant, so it makes sense to assess the overall pollution score. Moreover, this study suggested an inflection score of 86.9 that can be used as a control target. Nevertheless, we believe that this inflection point only applies to Han-Chinese people over 60 years.

Our current study also has some limitations. First, it lacked measurement data for assessing indoor air pollution, older adults are exposed to indoor air for more extended period. There is a significant difference in the concentration of pollutants between indoor and outdoor air, indoor air quality is influenced by the use of air purifiers, hoods, home structure, fuel, ventilation (33). Thus, the effects of these critical factors might be overlooked, next researches should investigate the relationship between indoor air and older adults' health. Second, due to the extensive range of individual activities, we did not precisely estimate the exposure level of each individual but only averaged the pollution values of the Binhai New Area to roughly estimate the exposure level of each patient. Additionally, the follow-up period of this study was short (2 years), and the lifetime-risk still needs to be studied. In the elderly cohort, bias may exist due to unequal numbers of age groups, with the older groups have risk of death. In our study, participants were required to participate at two follow-up visits. Essentially, this means that the dead individual has been removed. Next, we will consider the effects of death and use a competitive risk model to assess potential risk factors for CKD. Moreover, More confounding factors might need to be adjusted, such as consumption of alcohol, amount and duration of smoking, and type and duration of exercise. Finally, our current study showed the incidence rate was 24.8%, which maybe overestimated. However, when we use the CKD stage 3–5 as the outcome, a similar trend has been observed. CKD is common in older people and its prevalence increases in parallel with age (34). The prevalence of CKD in China and the United States were 34.6% and 31.5–32.9% (60–89 years) (35, 36). The population in the current study was based on adults over 60 years in China, they might be more susceptible to air pollution exposure. Hence, our results do not suitable for people under 60 years and non-Chinese.

## Conclusion

In summary, we demonstrated that both single and combined exposure to air pollutants was associated with an increased risk of CKD in the elderly. The air pollution score, PCA score and AQI were associated with risk of incident CKD in a dose-response relationship. This study would provide evidence for the development of environmental protection policies and emphasis the importance of persistent efforts to control air pollution.

## Data availability statement

The raw data supporting the conclusions of this article will be made available by the authors, without undue reservation.

## Ethics statement

The studies involving human participants were reviewed and approved by Chu Hsien-I Memorial Hospital of Tianjin Medical University. Written informed consent for participation was not required for this study in accordance with the national legislation and the institutional requirements.

## Author contributions

HYL and XS: methodology, software, formal analysis, visualization, and writing-original draft. PY: supervision, conceptualization, resources, writing-review and editing, project administration, and funding acquisition. XJ: data curation, validation, and investigation. SJZ, XJL, and YL: validation and investigation. PFB, JMC, FH, YZ, CL, and HL: investigation. ZC: formal analysis and writing-review and editing. All authors read and approved the final manuscript.

## Funding

This work was supported by the financial support from Tianjin Key Medical Discipline (Specialty) Construct Project (No. TJYXZDXK-032A), the Science and technology talent project of Tianjin Health Commission (No. RC20175), Scientific Research Program of Tianjin Education Commission (No. 2020KJ187), and Tianjin Science and Technology Plan Project Public Health Science and Technology Major Special Project (No. 21ZXGWSY00100). The funder was not for profit.

## Conflict of interest

The authors declare that the research was conducted in the absence of any commercial or financial relationships that could be construed as a potential conflict of interest.

## Publisher's note

All claims expressed in this article are solely those of the authors and do not necessarily represent those of their affiliated organizations, or those of the publisher, the editors and the reviewers. Any product that may be evaluated in this article, or claim that may be made by its manufacturer, is not guaranteed or endorsed by the publisher.

## Abbreviations

PM_2._5, particulate matter with an aerodynamic diameter lower than 2.5 μm. NO_2_, nitrogen dioxide. PM_10_, particulate matter with an aerodynamic diameter lower than 10 μm. AQI, air quality index. NO_2_, nitrogen oxide. SO_2_, sulfur dioxide. O_3_, Ozone. CO, carbon monoxide. FBG, Fasting blood glucose. HGB, hemoglobin. PLT, platelet. WBC, white blood cell. ALT, alanine transaminase. AST, aspartate transaminase. TC, total cholesterol. TG, triglyceride. BMI, body mass index. WC, waist circumference. SBP, systolic blood pressure. DBP, diastolic blood pressure. eGFR, estimated glomerular filtration rate. CKD: chronic kidney disease.
