# Supplementary material for: Joint exposure to outdoor ambient air pollutants and incident chronic kidney disease: A prospective cohort study with 90,032 older adults
Source: Front Public Health. 2022 Sep 15;10:992353. doi: 10.3389/fpubh.2022.992353 (PMC9524146; doi:10.3389/fpubh.2022.992353)

Supplementary files

Joint exposure to outdoor ambient air pollutants and incident chronic kidney disease: a prospective cohort study with 90,032 older adults

Inventory of Supplemental Information

I Introduction of the Tianjin Chronic Kidney Disease Study

II Supplementary Tables

Supplementary Table 1

Supplementary Table 2

III Supplementary Figures

Supplementary Figure 1

Supplementary Figure 2

Introduction of the Tianjin Chronic Kidney Disease Study

Cohort name and international clinical trial registration: Tianjin Chronic Disease Cohort Study; registration number, ChiCTR1900023701.

Project leader (lead institution): Pei Yu (Chu Hsien-I Memorial Hospital & Tianjin Institute of Endocrinology, Tianjin Medical University)

Project description: The study is an open cohort study that started in January 2013. It aims to explore the intrinsic patterns in the occurrence and progression of chronic diseases in the elderly population, identify key risk factors, and provide a scientific basis for effective prevention and control of the spread of chronic diseases.

Main objectives: Prediction, prevention and control strategy development of chronic diseases in the elderly

Study design: Open prospective cohort study in adult population.

Inclusion criteria: Adults aged 18 years or older.

Exclusion criteria: those who were disabled, semi-disabled, refused or unable to participate in regular medical check-up surveys.

Population characteristics: Cohort of adults aged 18 years or older from different levels of physical examination centers and communities in Binhai New Area, Tianjin. The population composition was matched for age, gender and occupation.

Completed participants per year/total number of participants: more than 300,000/2 million.

Starting and ending years and follow-up intervals: 2013 to present; at least 1 follow-up visit every 1 year.

Medical examination and measurement indicators: age, gender, smoking, alcohol consumption, occupation, education, blood pressure, heart rate, height, weight, waist circumference, ECG, abdominal ultrasound, blood routine (hemoglobin, platelets, lymphocytes, etc.), urine routine, liver and kidney function, electrolytes, fasting blood glucose, 2h postprandial blood glucose, triglycerides, total cholesterol, HDL, LDL, blood uric acid, albumin, glutamate transaminase, glutamic acid transaminase, etc.; exercise frequency, time and mode, dietary habits, etc.

Medication indicators: types of medications for various chronic diseases, medication dosage and frequency, compliance.

Outcome indicators: diabetes, hypertension, psychosis, hyperlipidemia, fatty liver, atrial fibrillation, disability and semi-disability, cardiovascular and cerebrovascular diseases, cancer, healthy life expectancy and all-cause mortality, etc.

Data collection: Experienced professional physicians and nurses from community and medical examination centers will complete data collection and fill in the system of Tianjin Community Health Service Center.

Data management: Uniformly uploaded to system of Tianjin Community Health Service Center for management, and data can be downloaded by logging into this system.

Supplementary Tables

Supplementary Table 1. The VIF of pollutants.

Variable	VIF	1/VIF
PM2.5	243.13	0.004113
CO	231.45	0.004321
SO2	180.64	0.005536
NO2	59.35	0.01685
PM10	41.27	0.024233
O3	27.54	0.036305

Supplementary Table 2. The principle components analysis of pollutants.

Variable	Comp1	Comp2	Comp3	Comp4	Comp5	Comp6
SO2	0.4123	-0.1462	0.5065	0.063	0.584	0.4551
PM2.5	0.4147	0.1372	-0.1004	0.3054	-0.634	0.5513
O3	-0.3948	0.8399	0.308	0.056	0.0674	0.1905
PM10	0.4054	0.414	-0.6862	-0.1576	0.4097	0.0255
NO2	0.41	0.1886	0.3439	-0.7389	-0.2909	-0.2179
CO	0.412	0.2179	0.2223	0.5734	0.0046	-0.636

Supplementary Table 3. Contribution proportion of each principal component.

Component	Eigenvalue	Difference	Proportion	Cumulative
Comp1	5.76198	5.6256	0.9603	0.9603
Comp2	0.136371	0.0771566	0.0227	0.9831
Comp3	0.059214	0.0240257	0.0099	0.9929
Comp4	0.0351883	0.029768	0.0059	0.9988
Comp5	0.00542038	0.00358914	0.0009	0.9997
Comp6	0.00183124	.	0.0003	1

Supplementary Figures

Supplementary Figure 1. Measurement of pollutants between 2017 and 2020.

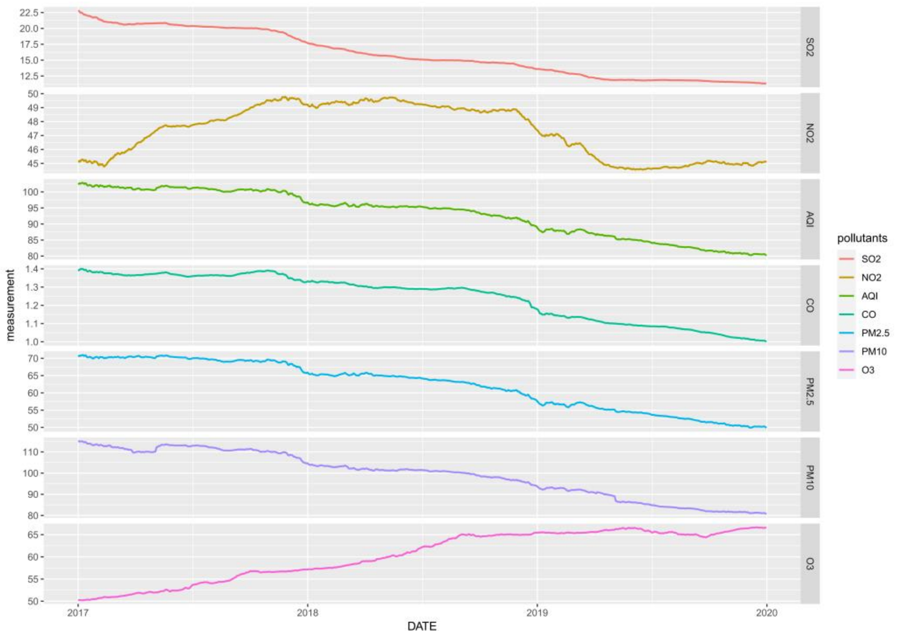

Supplementary Figure 2. ROC and calibration curves of AQI, combined score and PCA score.

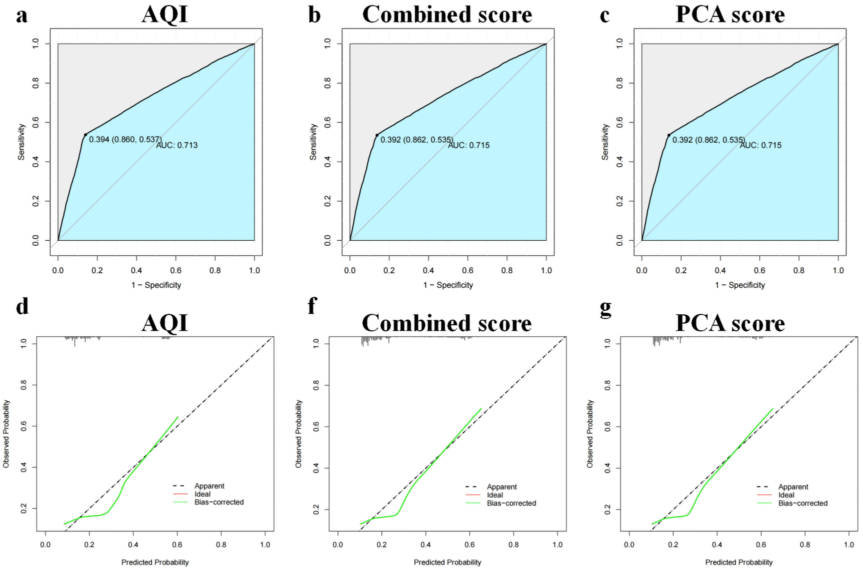

Supplement: Supplementary file 1 [file Data_Sheet_1.PDF]
